# Mobile Videoconferencing for Occupational Therapists’ Assessments of Patients’ Home Environments Prior to Hospital Discharge: Mixed Methods Feasibility and Comparative Study

**DOI:** 10.2196/24376

**Published:** 2022-07-05

**Authors:** Karine Latulippe, Dominique Giroux, Manon Guay, Dahlia Kairy, Claude Vincent, Katia Boivin, Ernesto Morales, Natasa Obradovic, Véronique Provencher

**Affiliations:** 1 Center for Interdisciplinary Research in Rehabilitation of Metropolitan Montreal Montréal, QC Canada; 2 School of Physical and Occupational Therapy McGill University Montreal, QC Canada; 3 Department of Rehabilitation Université Laval Quebec, QC Canada; 4 Centre de Recherche du Centre Hospitalier Universitaire de Québec Quebec, QC Canada; 5 Centre d'Excellence sur le Vieillissement de Québec Québec, QC Canada; 6 School of Rehabilitation Université de Sherbrooke Sherbrooke, QC Canada; 7 Research Center on Aging Sherbrooke, QC Canada; 8 School of Rehabilitation Université de Montréal Montréal, QC Canada; 9 Institut Universitaire sur la Réadaptation en Déficience Physique de Montréal Montréal, QC Canada; 10 Center for Interdisciplinary Research in Rehabilitation and Social Integration Quebec, QC Canada; 11 Centre Hospitalier Universitaire de Québec-Université Laval Quebec, QC Canada

**Keywords:** caregivers, feasibility, mixed methods, mobile videoconferencing, mobile phone, occupational therapy, discharge planning, home assessment

## Abstract

**Background:**

Occupational therapists who work in hospitals need to assess patients’ home environment in preparation for hospital discharge in order to provide recommendations (eg, technical aids) to support their independence and safety. Home visits increase performance in everyday activities and decrease the risk of falls; however, in some countries, home visits are rarely made prior to hospital discharge due to the cost and time involved. In most cases, occupational therapists rely on an interview with the patient or a caregiver to assess the home. The use of videoconferencing to assess patients’ home environments could be an innovative solution to allow better and more appropriate recommendations.

**Objective:**

The aim of this study was (1) to explore the added value of using mobile videoconferencing compared with standard procedure only and (2) to document the clinical feasibility of using mobile videoconferencing to assess patients’ home environments.

**Methods:**

Occupational therapists assessed home environments using, first, the standard procedure (interview), and then, videoconferencing (with the help of a family caregiver located in patients’ homes, using an electronic tablet). We used a concurrent mixed methods design. The occupational therapist's responsiveness to telehealth, time spent on assessment, patient’s occupational performance and satisfaction, and major events influencing the variables were collected as quantitative data. The perceptions of occupational therapists and family caregivers regarding the added value of using this method and the nature of changes made to recommendations as a result of the videoconference (if any) were collected as qualitative data, using questionnaires and semistructured interviews.

**Results:**

Eight triads (6 occupational therapists, 8 patients, and 8 caregivers) participated. The use of mobile videoconferencing generally led occupational therapists to modify the initial intervention plan (produced after the standard interview). Occupational therapists and caregivers perceived benefits in using mobile videoconferencing (eg, the ability to provide real-time comments or feedback), and they also perceived disadvantages (eg, videoconferencing requires additional time and greater availability of caregivers). Some occupational therapists believed that mobile videoconferencing added value to assessments, while others did not.

**Conclusions:**

The use of mobile videoconferencing in the context of hospital discharge planning has raised questions of clinical feasibility. Although mobile videoconferencing provides multiple benefits to hospital discharge, including more appropriate occupational therapist recommendations, time constraints made it more difficult to perceive the added value. However, with smartphone use, interdisciplinary team involvement, and patient participation in the videoconference visit, mobile videoconferencing can become an asset to hospital discharge planning.

**International Registered Report Identifier (IRRID):**

RR2-10.2196/11674

## Introduction

When planning hospital discharge, it is important to ensure that patients have optimal conditions for a safe return home and that patients’ care and services needs are met [1]. The occupational therapist can play an important role in achieving this objective [1] by providing recommendations (eg, technical aids, site planning, care services) to promote the patient’s autonomy and safety upon returning home. The home visit is a way for occupational therapists to obtain reliable information about the environment [2], which is essential for making recommendations that support the best fit between the person, their activities, and their environment. A randomized controlled trial [2] found that home predischarge assessment decreased the risk of falls, reduced the number of rehospitalizations, and increased the level of functional independence in patients with hip fractures. However, clinical (eg, patient fatigue or anxiety), organizational (eg, time available), and financial (eg, travel time and costs) constraints limit the implementation of home visits, despite their relevance [1-5].

Alternative means are currently used to assess the home environment when planning hospital discharge, such as interviews [6], consultation of home photos taken by caregivers [7], video [8], and virtual reality [9]. Interviews quickly give an idea of the environmental constraints perceived by the patient and caregivers. The interpretation of the occupational therapist is then based on this self-reported information, without having the opportunity to confirm it through direct observation [3]. Photos provided by the caregivers allow the occupational therapist to observe the patient’s environment [10]; however, this observation is dependent on the choice of photos and the angle used by the caregiver. Video also makes it possible to observe the environment [8]; however, similar to photos, it is an asynchronous means, and the occupational therapist’s observation is contingent upon what the relatives choose to show. Other methods such as virtual reality [9] and 3D photography [11,12] are currently being explored and are in the experimental stage [13].

Based on a growing body of literature, the use of mobile videoconferencing for remote rehabilitation interventions has potential clinical benefits [10,13,14]. By providing a detailed and real-time view of the home environment, mobile conferencing may help occupational therapists to improve the reliability of the data collected, which in turn guarantees appropriate recommendations. The occupational therapist, therefore, gives instructions to the caregiver who, using the electronic tablet, shows the facilities in the home for which more information is needed. However, empirical evidence is lacking to clinically support its use [15,16]. The aim of this study is (1) to explore the added value of using mobile videoconferencing compared with the standard procedure and (2) to document the clinical feasibility of using an electronic tablet to assess the patient’s home environment through videoconferencing.

## Methods

### Design

**Figure 1:**
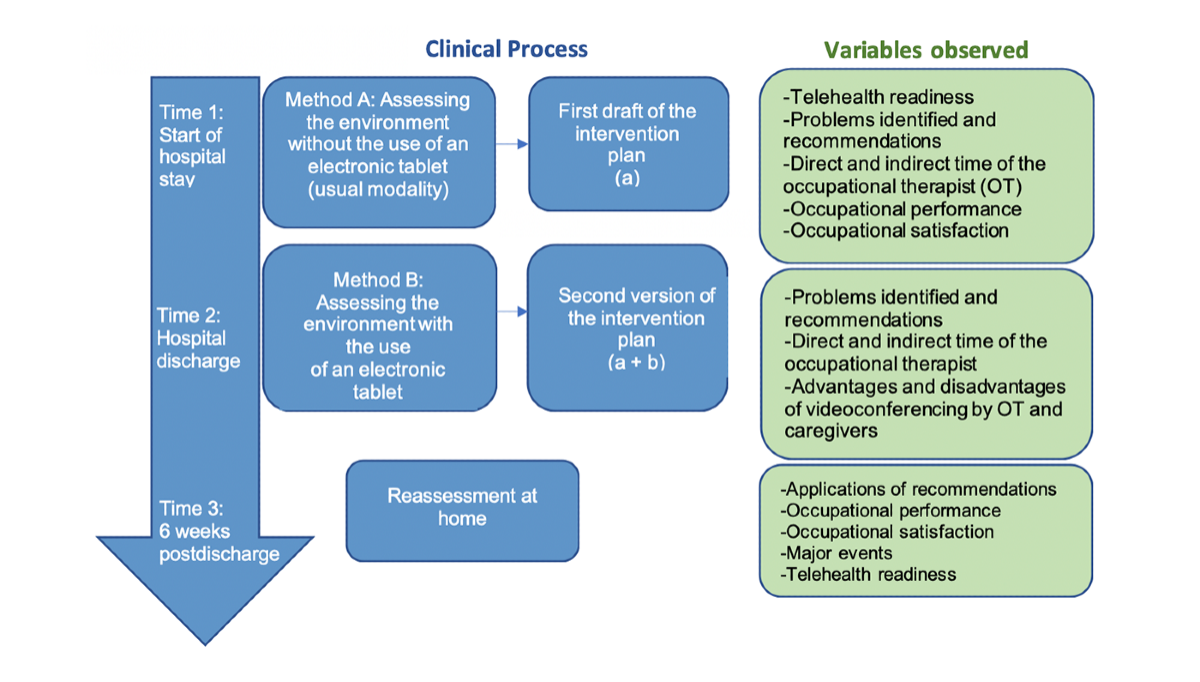
Study flowchart. OT: occupational therapist.

The methods used for this study are detailed in a published protocol [17]. We conducted a concurrent mixed methods feasibility study to compare 2 home assessment methods (Figure 1). In method A, information about the home environment was collected during an interview with the patient. In method B, evaluation of the home was carried out through mobile videoconferencing using an electronic tablet. For the videoconferencing evaluation, some occupational therapists used their work computer (when the facility allowed the installation of Skype for Business and a webcam was available), others, as well as the patients, used an HP elite pad and iPad tablet with a 3G mobile connection. An electronic tablet was provided to each caregiver with the exception of one caregiver who chose to use her own smartphone. Skype for Business was used for videoconferencing. The home assessment was conducted from the hospital center. The 2 assessment methods were compared to highlight the contribution of mobile videoconferencing to the standard evaluation (*A* versus *A and B*).

### Sampling and Recruitment

A purposeful sampling strategy was used in 2 regional hospitals in the province of Quebec, Canada. A triad consisted of (1) an adult patient with a loss of functional autonomy mainly due to physical disability, (2) their caregiver, and (3) the occupational therapist who conducted the assessment. The eligibility criteria for patients were (1) being hospitalized at the time of recruitment and (2) having a return home (including retirement homes) planned. Caregivers had to be able to (1) clearly express themselves orally (in French or English) and (2) walk without technical assistance. The occupational therapist had to have at least one year of clinical experience. Patients were excluded if they (1) had regular home monitoring by an occupational therapist in the community prior to hospitalization and (2) were unable to express themselves in a functional manner. The initial target sample was 18 triads (8 occupational therapists and 18 patient-related dyads).

### Data Collection Methods

Qualitative and quantitative data were collected in parallel.

#### Quantitative Data

We collected data on (1) occupational therapists’ receptivity to the use of mobile videoconferencing, using the French-Canadian version of the practitioner and organizational telehealth readiness assessment tools, for which a score >80 indicates that practitioners are well positioned to use telehealth, a score from 60 to 80 indicates that there are factors or elements that may negatively affect telehealth use, and a score <60 indicates that there are barriers to successful telehealth use by practitioners [18]; (2) the time spent evaluating the environment at the time of discharge (discussions, making an appointment with the caregiver, explanations prior to the assessment) using each method (with and without mobile videoconferencing), which was estimated by the occupational therapist; (3) major events after discharge, which were documented with the Social Readjustment Rating Questionnaire [19]; and (4) patient occupational performance and satisfaction was measured using the Canadian Professional Performance Measurement [20].

#### Qualitative Data

We also collected data on (1) the advantages and disadvantages of using mobile videoconferencing (the individual and semistructured interview guides—occupational therapists and caregivers’ versions—addressed previous and current experience with mobile videoconferencing use, the barriers and problems encountered with the use of mobile videoconferencing in the study, and the perceived benefits of adding this method); (2) occupational therapist’s professional recommendations, intervention plan, and the follow-up, which were charted using a pretested grid; and (3) the relevance and application of the recommendations, which were documented using a monitoring grid during an interview with the patient, with questions regarding the level of appreciation and barriers to implementation of the recommendations (approximately 20 minutes).

### Study Process

A participant’s guide was developed for the research assistant and occupational therapists. First, a research assistant invited the occupational therapists (in person or by telephone) to sign the consent form and to complete the French-Canadian version of the practitioner and organizational telehealth readiness assessment tools [18] and sociodemographic data form.

Second, the health care teams and occupational therapists identified patients who would potentially benefit from a home assessment prior to hospital discharge. These patients were offered the opportunity to participate in the study, and occupational therapists made sure to specify that a refusal would affect neither access to nor the quality of their assessment. If patients decided to participate in the study, the research assistant made an appointment with the patient and their caregiver to discuss the study in order to obtain informed consent and verify that the inclusion and exclusion criteria were met. Subsequently, the research assistant (who also has background in occupational therapy) conducted the Canadian Professional Performance Measurement [20] and collected sociodemographic data.

The occupational therapist conducted the home assessment by interviewing the patient and caregiver (method A). The occupational therapist recorded the time (direct, when the patient and the caregiver were physically or remotely present, and indirect, when the patient and the caregiver were not present) that it took to complete method A and documented any problems identified and recommendations (the first draft of intervention plan).

Next, the occupational therapist made an appointment with the caregiver for the home assessment via mobile videoconferencing (method B), which included the time necessary to pick up the electronic tablet and to teach the caregiver how to use the device. The occupational therapist recorded the time (direct and indirect) that it took to complete method B and modified the intervention plan where relevant.

Finally, the research assistant conducted a semistructured interview with the occupational therapist to identify prior and current experience with mobile videoconferencing, the barriers and problems encountered with the use of mobile videoconferencing in the study, and the perceived benefit of incorporating this method. The occupational therapist also completed the French-Canadian version of the practitioner and organizational telehealth readiness assessment tools [18] a second time. Six weeks after hospital discharge, the research assistant went to the patient's home. In the presence of the caregiver, the research assistant completed the Canadian Professional Performance Measurement [20] again, as well as the Social Readjustment Rating Scale [19]. She also conducted a semistructured interview related to the patient's satisfaction and the applicability of the recommendations that had been given at the time of discharge.

### Training

Occupational therapists were not formally trained in the study procedure; however, a step-by-step guide was provided on how to use the electronic tablet and videoconferencing app. A research assistant was available to answer questions and provide further guidance as needed.

### Analysis

Descriptive analyses were conducted for the receptivity scores collected from occupational therapists at the beginning (before the first patient) and at the end of the study (after the last patient), patient satisfaction and performance, as well as the type and number of unplanned events (confounding variables). We compared the recommendations from method A with those from the combination of methods A and B by identifying the differences and the nature of the differences (addition, modification). Finally, the application of the occupational therapist’s recommendations (methods A and B) by the patient was also evaluated 6 weeks after hospital discharge. Semistructured interviews were conducted, in which the perceived benefits and barriers of mobile videoconferencing were discussed, recorded, and transcribed verbatim. Using analytical questioning [21], the transcriptions were categorized by theme and analyzed by group (interview with occupational therapists, interview with caregivers, and occupational therapist’s professional recommendations). We used MAXQDA software (version 2018.1; Verbi GmbH) for analyses. Quantitative and qualitative data were integrated based on 2 analytical questions related to the study objectives: Which results inform us about the added value (or absence thereof) of mobile videoconferencing? Which results inform us about the clinical feasibility of using mobile videoconferencing for the purpose of home environment assessment before hospital discharge?

### Ethics Approval

The project was approved by the Research Ethics Committee of the Centre intégré universitaire de santé et de services sociaux de l’Estrie – Centre hospitalier universitaire de Sherbrooke (MP-31-2017-1485) and the Research Ethics Committee of the Quebec University Hospital – Université Laval (2017-3047). Mobile videoconferences were not recorded. Aside from the occupational therapists who performed the home assessment, no one could observe the patient’s home.

## Results

### Participants

Eight triads (6 occupational therapists, 8 patients, and 8 caregivers) were enrolled between April 2017 and April 2019 (Table 1).

The number of triads originally targeted was not reached. To better understand the issues surrounding patient recruitment and the feasibility of using mobile videoconferencing for home assessment, the research team decided to add open questions to the receptivity questionnaire (1) for occupational therapists who used mobile videoconferencing with at least one patient (n=6) and (2) for occupational therapists who participated in the project but who did not recruit patients (n=7) (Multimedia Appendix 1). In 1 instance, the mobile videoconferencing could not be used due to the absence of internet coverage in the municipality; the occupational therapist used video as an alternative. In addition, 1 patient could not be reached 6 weeks after discharge. (The occupational therapist who followed this patient thinks that he may have relocated to a different province.)

**Table 1:** Participant characteristics.

Group and characteristic					Value (n=8)
**Participants (n=8)**					
	**Age (years)**				
		Mean	79.5		
		Range	68-90		
	**Sex, n (%)**				
		Female	4 (50)		
		Male	4 (50)		
	**In-hospital stay (days)**				
		Mean	39		
		Range	10-96		
	**Principal diagnosis leading to hospitalization, n**				
		Infectious	1		
		Orthopedics	6		
		Neurology	1		
	**Medical complications, n**				
		Delirium	1		
		Postop shock	1		
	Comorbidities, n			8	
**Caregivers (n=8)**					
	**Age (years)**				
		Mean	58		
		Range	36-80^a^		
	**Sex, n (%)**				
		Female	7 (88)		
		Male	1 (12)		
	**Relationship to the patient, n**				
		Spouse	3		
		Child	3		
		Sibling	1		
	**Familiarity level with technology, n**				
		Poor	5		
		Average	1		
		Good	2		
**Occupational therapists (n=6)**					
	**Sex, n (%)**				
		Female	6 (100)		
		Male	0 (0)		
	**Overall work experience (years)**				
		Mean	8		
		Range	1-13		
	**Program work experience (years)**				
		Mean	6		
		Range	0.5-12		

^a^n=7, one value is missing.

### Difference Between Recommendations Before and After Mobile Videoconferencing

The majority of recommendations made before mobile videoconferencing (n=28) remained applicable afterward (n=25), except for 3 recommendations. Observation of the home environment through mobile videoconferencing made it possible to identify the lack of space required in the room to implement the planned recommendation and some incorrect information gathered during the interview (taps on the right side of the bathtub rather than on the left). These observations led to changes in the intervention plan.

Of the 8 patients, 7 patients’ initial intervention plans were modified after videoconferencing (Table 2). The changes were aimed at improving the person’s autonomy and safety, reducing the risk of falling, offering a cheaper or simpler solution than the one initially planned, and revising the initial recommendation in light of new information about the home environment that was not discussed during the interview. Occupational therapists also revised their recommendations for a better fit between the patient, their occupation, and the environment. Finally, 4 recommendations were completely modified as a result of videoconferencing—in 3 instances because the initial recommendation was not applicable and, in a fourth instance, because observing the environment made it possible to consider a return home following rehabilitation at an intensive functional rehabilitation unit instead of relocating the patient to a seniors’ residence, as recommended initially. In one instance, the intervention plan was not modified after mobile videoconferencing.

**Table 2:** Modifications of initial intervention plans after mobile videoconferencing.

Type and reason			Changes (n=18), n		Example
**Adding recommendations**					
	Optimizing the person’s autonomy	1		The installation of a support bar allowed the patient to transfer on her own rather than accompanied as in the initial recommendation	
	Optimizing safety and reducing the risk of falling	2		Adding a grab bar and stool in the shower to maximize safety	
	Offering a cheaper and simpler solution	1		Adding a grab bar to the wall instead of a toilet support frame as in the original recommendation.	
	Adjusting to new information that was not discussed at the interview	6		Observation of the environment identified 2 potential places for taking meals (a high table with stools and a standard table and chairs in the dining room); due to physical difficulties, using a stool was not recommended	
**Precision of recommendations**					
	Ensuring a better match between the patient, the patient’s occupation, and the environment	4		Precision about the orientation of the shower bench and support bars initially recommended	
**Change of recommendations**					
	The original recommendation was not applicable	3		The safety support on the righthand side is irrelevant given the countertop at an adequate height to the right of the toilet and the lack of space to install the grab bar on that side	
	Viewing the environment led to a return home	1		Once the environment is seen, there appeared to be no major architectural barrier to a return home if the patient manages to regain autonomy in her transfers and travel with the help of accessories	

### Perceived Benefits of Using Mobile Videoconferencing to Conduct a Remote Home Assessment

Overall, participants appreciated the use of the tablet and felt that “it adds something” (occupational therapist 6) to the standard evaluation. Specifically, caregivers perceived that the use of mobile videoconferencing allowed occupational therapists to obtain more precise information (Table 3).

According to participants, data collected from interviews can be wrong or incomplete because the caregiver neglects to take into consideration certain aspects. In fact, mobile videoconferencing induced modification of recommendations, such as correcting the provision for assistive devices to match the patient’s environment.

Yes, in fact the lady had given me inaccurate information about where the bath faucet was located, it was on the opposite side. I recommended a transfer bench with the handle on the wrong side. So, I adjusted that.

Occupational therapist 3

Another advantage of videoconferencing perceived by participants and occupational therapists was the opportunity for therapists to ask questions and provide feedback to the caregiver in real time. Caregivers felt guided in the assessment and identification of measures required, and the occupational therapists were able to document patients’ lifestyles and which elements of the environment they wanted to see.

I watched her take some measurements, some of which I may not have thought of not knowing [what the environment was] but since I was watching her, I was able to ask her to measure this and that. It’s great, I made a diagram. Seeing what she was doing was of great help to me.

Occupational therapist 4

Mobile videoconferencing was useful for estimating distances between various elements in the home environment. In addition, caregivers said that the videoconference visit reassured them. All caregivers mentioned that the mobile videoconferencing experience had been positive despite the fact that some encountered a few technical glitches.

**Table 3:** Main advantages and disadvantages associated with the use of mobile videoconferencing by occupational therapists and caregivers.

Characteristic			Description
**Advantages**			
	Common to occupational therapists and caregivers	Ability to make comments or provide feedback in real time Confirming the information obtained by the patient and caregivers Providing additional information on the patient’s lifestyle Ensuring the best choice of equipment Making sure that the caregiver is taking the right measurements and reassuring them about how they are doing Seeing the general condition of the environment (eg, cleanliness) Avoiding travel expenses and time	
	For occupational therapists	Discovering unanticipated barriers Dissipating remaining doubt and avoiding mistakes The involvement of the caregiver helps the patient to remember the recommendations Improving communication between the occupational therapist and the caregiver Promoting discussion between the occupational therapist and the patient if the latter participates in mobile videoconferencing For patients transferred to the intensive functional rehabilitation unit, mobile videoconferencing makes it possible to specify the rehabilitation objectives Seeing details and offering more specific recommendations Determining the pertinence of a home visit by the CLSC^a^ occupational therapist	
	For caregivers	Allowing the occupational therapist to identify problems that the caregiver had not thought of Feeling guided in the return home process Allowing patients to reconnect with their home and reflect on their return home Avoiding the need to explain everything Providing recommendations that don’t need tweaking Reassuring the caregiver	
**Disadvantages**			
	Common to occupational therapists and caregivers	Videoconferencing requires being comfortable with technology Videoconferencing requires additional time and availability of caregivers Videoconferencing constitutes additional stress for caregivers who are uncomfortable with taking measurements or using the tablet	
	For occupational therapists	Inability to observe the interaction between person, occupation, and environment No overview such as during a home visit in person Inaccessible if there is no Internet coverage in the municipality More time consuming than an interview	
	For caregivers	—^b^	

^a^CLSC: centres locaux de services communautaires (local community service centers).

^b^There were no other perceived disadvantages.

### Disadvantages of Using Mobile Videoconferencing to Conduct a Remote Home Assessment

One occupational therapist reported that mobile videoconferencing does not show the interaction between the person, the environment, and the person’s activities. In addition, mobile videoconferencing requires more time than interview assessment. Five caregivers mentioned that there were no disadvantages to mobile videoconferencing. The concerns brought up by caregivers were the same as those identified by occupational therapists (ie, stress of having to take measurements or use a tablet, especially if they are not comfortable with the device). Caregivers also highlighted the extra time that it took to pick up the equipment, undergo training, and conduct the videoconference.

### Perception of the Added Value of Mobile Videoconferencing

Mobile videoconferencing provided added value according to 3 of 5 occupational therapists. These 3 occupational therapists expressed readiness to promote the use of mobile videoconferencing to their peers.

Mobile videoconferencing takes longer to complete, but the recommendations are more specific. The ratio of time to what mobile videoconferencing requires in terms of logistics versus what it provides in terms of intervention offers added value.

Occupational therapist 3

For (another) patient, it helped define specific goals for her rehabilitation. It ensures that the recommendations are correct and feasible. Occupational therapists are often told that recommendations don’t work. Mobile videoconferencing is not pertinent in all cases but when it applies, it really offers added value. It applies when the occupational therapist has doubts about what the patient said.

Occupational therapist 4

Another occupational therapist, who was part of 2 triads, also failed to perceive any added value associated with the use of mobile videoconferencing.

The changes that the mobile videoconferencing made to the intervention plan were not essential to leave. They were aimed more at optimizing safety and could have been done by the occupational therapist at home. Although the mobile videoconferencing is more concrete than the interview and interesting, the time invested, and the minor changes made to the intervention plan mean that there is no added value.

Occupational therapist 5

### Changes in Satisfaction and Occupational Performance

There was a clinical difference between hospital stay and postdischarge patient performance scores (hospital: mean 4.0, SD 2.7; postdischarge: mean 6.2, SD 2.8) and satisfaction scores (hospital: mean 4.1, SD 3.1; postdischarge: mean 7.1, SD 2.1). A change of 2 points is considered a clinically relevant improvement or deterioration [22].

### Time Required for Mobile Videoconferencing

The mean direct time that occupational therapists reported having spent evaluating the environment through videoconferencing at the time of discharge (discussions, making an appointment with the caregiver, providing explanations prior to the assessment) was 104 minutes (SD 74). The mean indirect time (environment evaluation) was 64 minutes (SD 87).

### Occupational Therapists’ Receptivity to Mobile Videoconferencing

Assessment of the receptivity of occupational therapists who had recruited at least one patient indicated that there were barriers to successful telehealth use by practitioners (Table 4).

**Table 4:** Receptivity of occupational therapists who recruited at least one patient.

French-Canadian version of the Practitioner and Organizational Telehealth Readiness Assessment section			Score, mean (SD)		
			Before intervention (n=6)		Postdischarge (n=5)
**Total score (out of 85)**			51 (10)		56 (9)
	In order to meet the requirements for core readiness (out of 15)	7 (1)		8 (2)	
	In order to meet the requirements for engagement readiness (out of 35)	25 (3)		26 (3)	
	In order to meet the requirements for structural readiness (out of 35)	20 (7)		22 (5)	

### Factors Influencing the Choice to Use Mobile Videoconferencing

Several factors appeared to influence whether or not mobile videoconferencing was used by occupational therapists who recruited at least one participant.

One occupational therapist mentioned that, due to her workload, she could not always prioritize mobile videoconferencing over other tasks and did not always have time to do it. The necessary caregiver training on mobile videoconferencing also added to the time constraints associated with this method. Consequently, the occupational therapist’s perception of the time that training would take, dependent on whether or not the caregiver was comfortable with the technology, influenced their choice. According to the occupational therapists, meetings with caregivers to introduce mobile videoconferencing, scheduling virtual visits, and material recovery added to their workload, as well as, that of caregivers. Some occupational therapists doubted their ability to teach caregivers how to use mobile videoconferencing, insofar as this required availability, motivation, and collaboration. As reported by occupational therapists, a number of families refused to engage in mobile videoconferencing because the process seemed too cumbersome. However, for 6 of the caregivers, logistics were not a problem. In addition, some of the occupational therapists felt that meetings with caregivers involved discussions that went beyond mobile videoconferencing and therefore, in a context of limited time, this aspect is a challenge in terms of feasibility.

Sometimes it’s harder to get someone to go film or have a caregiver who is in step with current technologies.

Occupational therapist 3

You see the person, you do not just fix it and then move on to something else … she told me a lot of things and then they also have difficult things to do with them personally.

Occupational therapist 1

Occupational therapists sometimes anticipated the fact that the patient would be discharged from hospital before they had time to do the mobile videoconferencing or that it is not pertinent in view of the patient’s transfer to a rehabilitation unit. Finally, occupational therapists’ daytime work schedule did not match the availability of some caregivers, in which case, mobile videoconferencing was not considered.

### Caregiver Level of Comfort With Technology and Mobile Videoconferencing Training

Based on their own perceptions, caregivers’ comfort level with technology was poor (n=2), moderate (n=4), and good (n=2). Most felt that the training offered by the occupational therapist and the 2-page instruction booklet they were given helped them to learn how to use the tablet.

I was afraid I might not to be able to do it, but with that short training, it seemed simple enough and I enjoyed trying to help.

Caregiver 5

For one caregiver, however, the training was not sufficient. This caregiver used help from a third party (siblings) during the videoconference. Two other caregivers received help from a third, although their levels of comfort with the technology were moderate and high.

It didn’t take long; the hardest part was to learn how to operate the tablet and finally it was my sister who turned it on because I had already forgotten how it worked...I’m not used to that myself.

Caregiver 2

### Perceptions of the Relevance of Home Assessment, Mobile Videoconferencing, and Recruitment Difficulties

#### Relevance of Home Assessment in Hospital Discharge

Of the occupational therapists who participated in the project but did not recruit patients, 5 occupational therapists considered home assessment prior to hospital discharge to be pertinent.

It’s important for the safety of the patient and in the prevention of falls, in the maintenance of autonomy also.

Occupational therapist G

It’s an integral part of my job.

Occupational therapist B

However, 2 occupational therapists believed that it was the community occupational therapist and not the hospital occupational therapist’s role to do the home assessment.

I think it’s the role of the occupational therapist at the CLSC [centres locaux de services communautaires (local community service center)] to do the home assessment because she has that expertise.

Occupational therapist D

#### Trust in the Interview as a Home Assessment Method

According to most occupational therapists, the amount of trust that can be placed in an interview method depends on the patient. If the patient has no cognitive impairments and the family confirms the information, then it can be relied upon. Conversely, the method cannot be used with patients who have impaired memory or difficulty expressing themselves. The method is even less reliable if a caregiver is not present, which was mentioned by one occupational therapist, who also stressed the possible discrepancy between patient, patient family, and professional perceptions.

We are confused by the patient’s speech. For example, the patient considers that his home allows to circulate with a walker while a professional would judge the opposite following assessment.

Occupational therapist C

In the opinion of some occupational therapists, when doubt exists, photos can be requested from the family or a referral sent to the CLSC occupational therapist. However, there may be a significant delay if the home assessment is done by the CLSC occupational therapist due to their own workload.

#### Prerequisites for the Use of Mobile Videoconferencing by Occupational Therapists

Many occupational therapists (n=3) commented that they did not have the necessary prerequisites to use mobile videoconferencing (ie, good knowledge of how to use the tablet, ability to solve technical contingencies, and ability to teach the family how to use it). One occupational therapist believed that with good training she could manage. The others felt comfortable using mobile videoconferencing (n=3).

#### Profile of Patients Who Could Benefit From Mobile Videoconferencing

According to occupational therapists, the patients who would benefit from mobile videoconferencing are patients who have permanent motor disorders, who are already known to the therapist, who are young adults, who are alone, who need a walker in the home, who have cognitive impairments and need to be tested in conditions that are similar to what they are used to, or whose entourage is comfortable with technology and available. Some occupational therapists said that this patient profile is quite common in practical settings, while others disagreed.

#### Reasons for Nonrecruitment

Finally, in order to explain the reasons why they were unable to recruit patients in the context of the project, occupational therapists mentioned the movement of staff, the impression that it would be asking too much of the caregiver, the lack of time, the difficulty of coordinating the availability of caregivers with their own, having caregivers at ease with technology, the perception of duplicating work with the CLSC, thinking of recruiting patients, having patients who correspond to the inclusion criteria, and work overload.

### Problems Encountered With the Use of Mobile Videoconferencing

Some technical problems were encountered during the study. Communication with the clinician was generally adequate. The sound and the image were judged to be clear by all the participants. With the exception of the lack of internet coverage in the municipality where one patient resided, the technical problems did not prevent the use of mobile videoconferencing or the home evaluation and were not raised as being inconvenient for patients using mobile videoconferencing.

### Participant Suggestions on Improving Home Assessment Using Mobile Videoconferencing

One occupational therapist suggested that it would be useful to record the videoconference visit and subsequently review the assessment (as needed or depending on the patient’s progress). A caregiver also recommended that the videoconference visit be recorded and available to other professionals. She was again asked about her environment in the rehabilitation unit following her stay at the unit where the initial assessment took place and felt that she was duplicating what had already been done. Another occupational therapist suggested that the interdisciplinary team should be involved in the videoconference. First responders, often the social worker, could explore the possibility of doing the mobile videoconferencing with the patient’s family even before the occupational therapist receives the referral in order to address the time constraints of short stay. In addition, it may be pertinent for the physiotherapist to see the walking distances between the home and the parking lot and inside the home, and for the social worker to verify the safety and cleanliness of the home environment. Finally, one occupational therapist conducted the mobile videoconference together with the patient. She explained that the patient was able to provide details of her lifestyle and this experience motivated her in her rehabilitation because she was able to visualize what her return home would be like. This occupational therapist recommended patient participation in mobile videoconferencing.

## Discussion

### Principal Results

The use of mobile videoconferencing after the interview generally led occupational therapists to modify their initial intervention plan. Most changes were considered by occupational therapists to be minor inasmuch as they were expected to have little impact on a safe return home. However, 3 assistive devices recommended after the interview raised some issues after discharge. In addition, based on mobile videoconferencing, the decision of the interdisciplinary team and that of the patient himself to transfer to a seniors' residence was modified, and the patient returned home upon discharge instead. This is a clinically important point. Unimplementable recommendations (such as the 3 assistive devices mentioned above) can interfere with older adults’ ability to age in their homes, and a change in home environment is no small matter in a person’s life.

Overall, the perceived advantages of mobile videoconferencing for occupational therapists and caregivers exceeded the disadvantages; however, the nature of the disadvantages—time required to conduct mobile videoconferencing (meeting planning, tablet training, equipment loan, virtual visit) combined with the increased workload perceived by occupational therapists, intervention priorities such as pressure injury, availability of caregivers on working hours, and the short length of stay—do not support its use. More specifically, the perceived reliability of data collected through interviews and the short time required for interviews led occupational therapists to prefer interviews as an evaluation method. This is consistent with the conclusions from a scoping review [13] on the use of information and communication technology for home assessment. Our study highlighted that mobile videoconferencing is considered beneficial by occupational therapists when the patient has a cognitive impairment and a caregiver is not available, both of which reduce the reliability of data collected through interviews. However, for individuals with cognitive impairment, it is very important to observe their interaction with their home environment, and mobile videoconferencing used in the manner described in this study does not allow for this interaction to be seen [23]. Also, in our study, availability and motivation of caregivers were identified as prerequisites for the use of mobile videoconferencing by occupational therapists.

Another clinically relevant finding was that mobile videoconferencing required increased involvement on the part of caregivers in discharge planning. This appears to be an advantage for improving communication between the clinician and caregivers, thereby increasing the probability that the caregiver will implement the occupational therapist's recommendations. In contrast, some occupational therapists, including those who did not recruit a patient, felt uncomfortable burdening caregivers with this task. In fact, some eligible patients were not part of the study because the caregiver declined to participate due to their busy schedule. Knowing that caregivers are at risk of exhaustion [24], clinicians may have been reluctant to add to their burden of care. The family caregivers enrolled in the study, who may arguably be more available and interested in the project, commented that the logistics surrounding mobile videoconferencing had not been a problem. They said that the mobile videoconferencing had reassured them and that they appreciated being guided by the occupational therapist to make the measurements. Holland and colleagues [25] reported that seeing the clinicians on video made caregivers feel as if they were at home with them, which facilitated interactions. Chi and Dimiris [26] also found that caregivers felt more involved in the process. Therefore, mobile videoconferencing can be perceived as a burden by some caregivers and as a facilitator by others.

Some feasibility issues may explain recruitment difficulties and, therefore, will have an impact on the choice to use mobile videoconferencing or not. Based on the Telehealth Readiness Assessment questionnaire [18], there was a degree of reluctance with respect to telehealth. These findings are not consistent with those of a study [27] in which clinicians were reported to be supportive of more frequent use of the telecommunication system. However, our results may be influenced by clinicians’ perceived openness of their workplace to telehealth. Indeed, in the Telehealth Readiness Assessment questionnaire [18], almost half of the points (40 out of 100) pertain to how clinicians perceived the receptivity of the institution. In one study [15], occupational therapists reported that they needed more training in communication technology use but organizational constraints were a barrier [15]. This is consistent with our finding that many occupational therapists did not have the skills to use mobile videoconferencing or to show caregivers how to use the technology. This perception of a lack of technological skills, combined with occupational therapists’ perceptions that caregivers who are less familiar with technology would require more time, may explain why they favored the involvement of caregivers who are familiar with the technology. Our conclusions are consistent with those of Ninnis and al [13], who suggested that therapists consider the use of mobile apps to be appropriate for some patients but not those who are less confident or less able to use new technologies. However, in our study. it does not appear that the caregivers’ level of comfort with the technology affected its use.

### Future Directions

Some occupational therapists and caregivers suggested that the use of caregivers' own smartphones, despite potential confidentiality issues, would allow for a better videoconferencing experience. Smartphones are becoming more and more popular among people aged 65 and over [28]. In addition to precluding the need for mobile videoconferencing training, the use of their own device would eliminate the need for caregivers to come to the hospital to pick up equipment. We are currently working with engineers on making personal smartphones safe and simple to use (only one button to press), with options to measure distances between home facilities through screenshots. Another suggestion made by one occupational therapist was to involve patients in the videoconference, which is in line with shared decision-making and patient-centered approaches [29-31]. The involvement of a social worker and physiotherapist could also help to gather further information (presence of an interior and exterior staircase for example) during the virtual visit and thus optimize hospital discharge planning (such as planning the need for assistance with mobility). We suggest that future studies compare standard assessment (interview), videoconference, and in-person visits of the home environment with the patient in terms of benefits and clinical, ethical, and financial issues [32,33]. It would also be of interest to document the clinical reasoning behind the decision whether or not to assess the home environment, through mobile videoconferencing or otherwise, in order to guide occupational therapists on the best methods to use for this and on how to best use their time [5].

### Limitations

This study has some limitations. First, we had fewer participants than desired. The recruitment difficulties encountered during the study underscore the need to make organizational changes to support the use of mobile videoconferencing in routine care. Nevertheless, the added value perceived by participants as well as the opportunity to obtain additional and more appropriate recommendations suggest the relevance of using mobile videoconferencing. Second, it would have been relevant to further document the occupational therapists’ and caregivers’ level of comfort with technology use in order to better understand how it influenced occupational therapists’ receptivity and participant recruitment. Occupational therapists were not asked to recruit the ideal candidate, but a participant selection bias cannot be excluded because of workload concerns. To reduce their workload, they may have been inclined to select patients with family caregivers who were comfortable with the technology or who were motivated to use videoconferencing. Moreover, the analysis was performed by one person (KL). However, the interviews were transcribed verbatim, and 2 co-authors who participated in the interviews (KB, MG) attested to the consistency between themes and interviews. Finally, the start of the COVID-19 pandemic occurred in the period between the study’s completion and its publication, which may also impact the results as the pandemic forced occupational therapists and the general population to learn about, if not improve, their technological proficiency and to use mobile video conferencing more frequently.

### Conclusions

Clinical feasibility issues were found when using mobile videoconferencing to support hospital discharge planning. Although mobile videoconferencing provides multiple benefits, such as more appropriate occupational therapist recommendations, the inconveniences, such as time constraints, make it difficult to perceive the added value of this method. However, it was suggested that having caregivers use their own smartphone, involvement of the interdisciplinary team, and patient participation in the videoconference would mitigate these inconveniences.

## Appendix

Multimedia Appendix 1 Added open questions to the receptivity questionnaire for occupational therapists who did not recruit a patient.

## Abbreviations

CLSC: centres locaux de services communautaires (local community service centers)
