# Supplementary material for: Mobile Videoconferencing for Occupational Therapists’ Assessments of Patients’ Home Environments Prior to Hospital Discharge: Mixed Methods Feasibility and Comparative Study
Source: JMIR Aging. 2022 Jul 5;5(3):e24376. doi: 10.2196/24376 (PMC9297141; doi:10.2196/24376)
Supplement: Multimedia Appendix 1 [file aging_v5i3e24376_app1.pdf]

Multimedia Appendix 1: Added open questions to the receptivity questionnaire for occupational therapists who did not recruit a patient.

-How do you see the appropriateness of conducting a home assessment for discharge planning from a hospital setting?

-How confident are you in the description of the home that your client and his or her family give you in an interview?

-What are the facilitators and barriers you encounter when you want to do a home assessment from the hospital?

-How do you feel about using mobile videoconferencing to conduct a home assessment? Do you see any added value compared to your usual practice?

-What do you think are the prerequisites for using mobile videoconferencing for home evaluation? Do you consider yourself to have these prerequisites?

-For which patient profile do you think mobile videoconferencing could be useful if required? How common is this profile in your work setting?

-With respect to your experience in the study, which of these barriers did you consider to be barriers to recruitment :
-Perception of time required
-Thinking about recruiting patients
-Perception that patients do not fit the desired profile
-Other
